# Development and feasibility of first- and third-person motor imagery for people with stroke living in the community

**DOI:** 10.1186/s40814-023-01263-9

**Published:** 2023-03-03

**Authors:** Nandana Welage, Michelle Bissett, Kristy Coxon, Kenneth N. K. Fong, Karen P. Y. Liu

**Affiliations:** 1 School of Health Sciences, Western Sydney University, Penrith, NSW 2751 Australia; 2 Present address: Department of Disability Studies, University of Kelaniya, Ragama, Sri Lanka; 3 Faculty of Health, Southern Cross University - Gold Coast Campus, Gold Coast QLD, Australia; 4 Translational Health Research Institute, Western Sydney University, Penrith, NSW Australia; 5 Department of Rehabilitation Sciences, The Hong Kong Polytechnic University, Hung Hom, Hong Kong SAR

**Keywords:** Mental imagery, Hand function, Stroke, Rehabilitation

## Abstract

**Background:**

Impairment of arm movement occurs in up to 85% of people post-stroke, affecting daily living activities, and quality of life. Mental imagery effectively enhances hand and daily function in people with stroke. Imagery can be performed when people imagine themselves completing the movement or imagine another person doing it. However, there is no report on the specific use of first-person and third-person imagery in stroke rehabilitation.

**Aims:**

To develop and assess the feasibility of the First-Person Mental Imagery (FPMI) and the Third-Person Mental Imagery (TPMI) programs to address the hand function of people with stroke living in the community.

**Methods:**

This study comprises phase 1—development of the FPMI and TPMI programs, and phase 2—pilot-testing of the intervention programs. The two programs were developed from existing literature and reviewed by an expert panel. Six participants with stroke, living in the community, participated in the pilot-testing of the FPMI and TPMI programs for 2 weeks. Feedback collected included the suitability of the eligibility criteria, therapist’s and participant’s adherence to intervention and instructions, appropriateness of the outcome measures, and completion of the intervention sessions within the specified time.

**Results:**

The FPMI and TPMI programs were developed based on previously established programs and included 12 hand tasks. The participants completed four 45-min sessions in 2 weeks. The treating therapist adhered to the program protocol and completed all the steps within the specified time frame. All hand tasks were suitable for adults with stroke. Participants followed the instructions given and engaged in imagery. The outcome measures selected were appropriate for the participants. Both programs showed a positive trend towards improvement in participants’ upper extremity and hand function and self-perceived performance in activities of daily living.

**Conclusions:**

The study provides preliminary evidence that these programs and outcome measures are feasible for implementation with adults with stroke living in the community. This study outlines a realistic plan for future trials in relation to participant recruitment, training of therapists on the intervention delivery, and the use of outcome measures.

**Trial registration:**

Title: Effectiveness of first-person and third-person motor imagery in relearning daily hand tasks for people with chronic stroke: a randomised controlled trial.

Registration No: SLCTR/2017/031. Date registered: 22nd September 2017.

**Supplementary Information:**

The online version contains supplementary material available at 10.1186/s40814-023-01263-9.

## Key messages regarding feasibility

- This study tested the viability of the First-Person Mental Imagery and the Third-Person Mental Imagery program programs before implementation in a randomized controlled trial.
- The findings showed that the participants’ eligibility criteria and outcome measures were suitable. Participants and therapists could adhere to the program, follow the instructions and complete the sessions within the specified time. Participants also showed an improving trend in the outcome measures used.
- The literature revealed the unique benefits of the two perspectives of imagery. This feasibility study provided experts’ opinions on the development of the two programs. The two programs are deemed feasible concerning the recruitment and assessment procedures for conducting a randomized controlled trial to investigate the effectiveness of the intervention.

## Background

Stroke is reported as the second or third most common cause of death [1]. It was reported that impairment of arm movement occurs in up to 85% of people post-stroke [2]. Impairment in arm and hand function may lead to a significant long-term impact on activities of daily living, work and leisure, and a corresponding decreased perception of quality of life [3]. Gradual improvement in arm and hand movement in people post-stroke is seen until at least 1 year post-hospital discharge [4]. Therefore, it is essential to address the arm and hand impairment of people with stroke living in the community. Motor imagery (MI) refers to mentally practising a task without physically performing it [5]. Evidence exists that MI, combined with actual task practice, can improve hand function in people with stroke [6]. Neuroimaging studies have shown that MI activates similar brain regions as occurs when people execute the actual movement [7]. Kho, Liu [6] reported that the benefits of MI, with activations of similar brain regions similar to actual movement, might allow people with stroke who could not physically move their limbs to have those brain regions stimulated.

There are two methods of practicing MI: First-Person Motor Imagery (FPMI) and Third-Person Motor Imagery (TPMI) [8–10]. In FPMI, the persons imagine performing the movement from inside their body [11]. In TPMI, the persons imagine performing the movement from the outside of their body or imagines someone else performing the movement [8, 9, 11, 12]. Individuals might feel more motivated to succeed on a future task when they visualize its successful completion from a third-person perspective [13]. Neuroimaging studies have identified that FPMI and TPMI share activations in both common and different cortical areas of the brain [14]. FPMI and TPMI were found to associate with activation in the motor areas such as the supplementary motor area and the precentral gyrus, the visual area such as the middle temporal/V5 complex, and the precuneus for memory tasks. In addition, FPMI might require more interpretation of sensory information and involved the right inferior parietal, precuneus, and somatosensory cortex.

In summary, the literature suggests that the FPMI may better resemble the actual motor performance while the TPMI may enhance motivation and self-perception in addition to motor functions. However, this proposition has not been adequately investigated in people with stroke. Depending on the needs of people with stroke and the purpose of rehabilitation, this research may provide information to assist clinicians in using the specific imagery perspective, which can further promote the rehabilitation outcome.

### Aims and objectives

This study aimed to test the viability of the FPMI and TPMI programs before implementation in a randomized controlled trial. The study developed two novel mental imagery intervention programs by reviewing the literature, collecting feedback, and obtaining consensus from an expert panel on critical parameters for people with stroke living in the community. The developed programs were pilot-tested for 2 weeks on six people with stroke living in the community.

## Methods

The study was conducted in two phases. Wight, Wimbush [15]’s steps in quality intervention development were adopted. Phase 1 included [1] defining and understanding the problem and existing interventions by a review of literature and [2] identifying parameters to modify, and adapting the intervention by collecting feedback from the experts who are the therapists delivering the intervention. Phase 2 involved testing the intervention protocols on people with stroke living in the community. The study combined published research evidence with the views and actions of people who would use the intervention programs [16].

Ethics approval was obtained from the Human Research Ethics Committee at the Western Sydney University (reference number H12057), Ethics Review Committees of the University of Kelaniya, Sri Lanka (reference number: P/154/06/2017), the National Hospital of Sri Lanka (reference number: AAJ/ETH/COM/2017–08) and Colombo South Teaching Hospital, Sri Lanka (reference number: AA/4/2017/596).

### Phase 1

#### Aim

The aim of the study is to develop the FPMI and TPMI intervention programs and collect feedback from an expert panel to review the viability of the intervention programs.

#### Methods

The first step in this stage involved the development of the intervention programs. We completed a systematic review to identify the mental imagery strategies previously used and applicable to people post-stroke [17]. This revealed the use of the first-person and the third-person imagery and 12 daily hand tasks. A draft program outline was developed.

The second step involved feedback from four experts in stroke rehabilitation in Sri Lanka on the viability of the intervention programs in Sri Lanka [16]. The experts were occupational therapists with 6 to 17 years of experience in stroke rehabilitation. The therapists were provided with the two intervention manuals, including the materials used such as the cue cards and video recordings, and were asked to rate and comment on (1) suitability of the intervention programs for adults with stroke living in the community, (2) appropriateness of the daily hand tasks used in the programs for adults with stroke, (3) appropriateness of the frequency and duration of the programs, and (5) clarity of instructions in the intervention programs. Panel members were asked to rate these five questions using a 5-point Likert scale where 1 = poor, 2 = fair, 3 = good, 4 = very good, and 5 = excellent. The expert panel review was conducted online. Panel members were requested to comment further if they provided 1 (poor) or 2 (fair) ratings. Based on the feedback received, the research team discussed and revised the programs. 

## Results

All four panel members were Sri Lankan occupational therapists who have had over five years of experience in the field of neurological rehabilitation and possessed a Bachelor’s degree in occupational therapy (Table 1).

**Table 1:** Expert panel

Type	*N* (%)	
Country of work	Sri Lanka	4 (100)
Sex	Female	4 (100)
Education	BSc	4 (100)
Experience in neurological rehabilitation	6 years	1 (25)
	8 years	2 (50)
	17 years	1 (25)

### Expert panel’s opinion

All questions received a median rating of 3 and above (good to excellent) for both the FPMI and TPMI programs. The lowest median rating of 3 (good) was found in frequency and duration of both programs. One therapist suggested allowing more time for the whole program. After reviewing findings from previous studies that showed positive effects after 6 weeks of MI training in patients with sub-acute stroke [10, 18], the program was adjusted to be delivered over 6 weeks.

### The FPMI and TPMI programs

The FPMI and TPMI intervention programs developed were based on previous programs designed for people with stroke [10, 19] and incorporated 12 daily hand tasks. In the FPMI program, the therapist would guide the participants to perform the task first, which would allow the participants to self-generate images of the task steps involved. In the TPMI program, video recordings of someone else performing the task would be shown to participants using a computer laptop, providing the prompt to generate and imagine the steps involved. The video recordings of the tasks lasted for less than 1 min. The frequency and duration of the FPMI and TPMI intervention programs were set following the standard occupational therapy practice in Sri Lanka and also similar to the schedule of two MI studies which demonstrated a significant improvement in the treatment groups [10, 20]. Both programs were to be conducted in a quiet clinic room free from distraction. The full programs included two intervention sessions a week for 6 weeks, with each session lasting 45 min in duration. Sessions of 45 min also matched with the duration of an occupational therapy session in public hospitals in Sri Lanka. Additional file 1: Appendix I provides further details about the intervention programs.

### Phase 2

#### Aim

To pilot-test the FPMI and TPMI programs and study protocols by reviewing (1) suitability of the eligibility criteria, (2) therapist’s and participant’s adherence to intervention, (3) participant’s ability to follow the instructions, and (4) participant’s completion of the intervention sessions within the specified time.

#### Methods

The feasibility and program outcomes of both intervention protocols were pilot-tested for 2 weeks (four sessions) using a pre- and post-program design. Six participants from the neurology clinics at two teaching hospitals in Colombo, Sri Lanka, were recruited by convenience sampling. Previous studies indicate that six participants is adequate to assess the feasibility of intervention programs [21]. Medical staff in the neurology clinics identified potential participants. Participants were invited to take part in this study via flyers. Written informed consent was obtained from all participants before involvement in the study. The assessment used in screening the potential participants are standardised with good reliability and validity.

#### Eligible participants

1. Were aged between 18 and 80 years
2. Had a diagnosis of hemiplegia due to stroke (infarct or haemorrhage)
3. Experienced a stroke more than 3 months prior to study enrolment (Bernhardt et al., 2017)
4. Resided in the community
5. Were able to complete advanced hand activities 1, 2, and 3 of Motor Assessment Scale (Carr et al., 1985)
6. Had a score of ≥ 24 points on the Mini-Mental State Exam [22]
7. Provided voluntary consent to participate in the study

People were excluded from the study if they had the following:

1. Visual and perceptual problems including hemianopia and unilateral neglect
2. Excessive pain in the affected arm as measured by a score of ≥ 4 on a 10-point Visual Analogue Scale [23]
3. Spasticity of ≥ 3 on the Modified Ashworth Scale [24]
4. Pre-existing musculoskeletal, neurological (apart from a stroke), or other conditions that may affect upper limb function

#### Feasibility of the FPMI and TPMI programs

Feasibility criteria of the intervention programs examined the (1) suitability of the eligibility criteria, (2) participant and therapist adherence to intervention, (3) ability of participants to follow the instructions, and (4) whether participants were able to complete the intervention sessions within the specified time. 

A research assistant, who was an occupational therapist with over 5 years of experience in the field of neurological rehabilitation and possessed a bachelor’s degree in occupational therapy, delivering the interventions and collected information regarding the feasibility criteria. This research assistant was a different person from the Phase 1 expert panel. The training was provided by the primary researcher in delivering the intervention.

#### Pilot-testing

Eight outcome measures were identified for the pilot study and were tested on the participants before and after the intervention. The primary researcher was the assessor and conducted all assessments. These outcome measures represented four domains of functions:

1. Performance-based upper extremity and hand function (Jebsen-Taylor Hand Function Test (JTHFT) [25], Fugl-Meyer Assessment of Motor Recovery after Stroke (FMA) [26], Nine Hole Peg Test (NHPT) [27], In-Hand Manipulation Assessment (IHMA) [28]).
2. Self-perceived upper extremity and hand function [Motor Activity Log (MAL) [29], with both Amount of Use (AOU) and Quality of Movement (QOM)].
3. Self-perceived daily functions (Lawton Instrumental Activities of Daily Living Scale (IADL) [30], Canadian Occupational Performance Measure (COPM) including performance (COPM-P) and satisfaction (COPM-S) [31]).
4. Quality of life (Stroke Specific Quality of Life Scale (SSQOL) [32]).

Information on the outcome measures is provided in Additional file 2: Appendix II. Data was collected before and after implementation of the intervention. The description of the participant’s responses to the study processes and outcomes are reported to support the clinical application.

## Results and discussion

Six participants (aged between 46 and 67 years) were recruited and completed the pilot-testing (three in the FPMI program and three in the TPMI programs). They ranged in. pseudonyms were assigned to the participants (Table 2).

**Table 2:** Participants’ demographic data

Participant	Age	Sex	Diagnosis	Duration of lesion	Intervention received
#1	62	Male	Left intra-cranial haemorrhage	6 months	FPMI
#2	52	Female	Left cortical infarction	28 months	FPMI
#3	67	Female	Right ischemic stroke	11 months	FPMI
#4	46	Female	Left fronto-parietal infarction	6 months	TPMI
#5	53	Female	Left ischemic stroke	12 months	TPMI
#6	60	Male	Left cortical infarction	9 months	TPMI

*Notes*. *FPMI* first-person motor imagery, *TPMI* third-person motor imagery

### Feasibility of the FPMI and TPMI programs

The treating therapist was able to adhere to the programs and completed all the steps in the FPMI and TPMI programs for each hand task within 15 min and three daily hand tasks in 45 min. It was identified that all hand tasks were suitable for adults with stroke. 

All participants were able to engage in the intervention program. They were also able to follow the instructions given by the treating therapist and engage in imagery from the first-person perspective for 8 min for each hand task without distraction. All participants reported that they were able to imagine the task “clearly and reasonably vividly” during the task practice. Two participants reported dryness of mouth during the thinking aloud component. Providing a glass of water for participants was used to address this problem. During the feedback, when viewing the video recording, a participant commented that keeping the video footage about 2 ft. away would be suitable.

The above information showed that the selection criteria were appropriate. Participants had the required hand function to practise the hand tasks for the time necessary, they had the cognitive ability to follow instructions, perform imagery and complete all the tasks.

### Pilot testing

While the clinical significance of the change of scores could not be established with only the pilot testing of four intervention sessions, individual results of the three cases showed a positive trend towards improvement in the performance-based and self-perceived upper extremity and hand function. All participants, except Participant #3 (TPMI), showed improvement in self-perceived daily functions. This 60-year-old male with a right-sided stroke did not show improvement in IADLS. However, COPM-P and COPM-S, which measured self-perceived daily functions, showed a slight improvement in all three participants.

Participant #6, the oldest participant in the TPMI group (60 years) who experienced his stroke 9 months ago, might be functioning at a stable level. Therefore, instrumental activities such as shopping, laundry, transport, and food preparation would not show a remarkable improvement with only 2 weeks of MI practice.

The results of the six participants are overviewed below.

#### Participant #1 (completed the FPMI program)

**Fig. 1:**
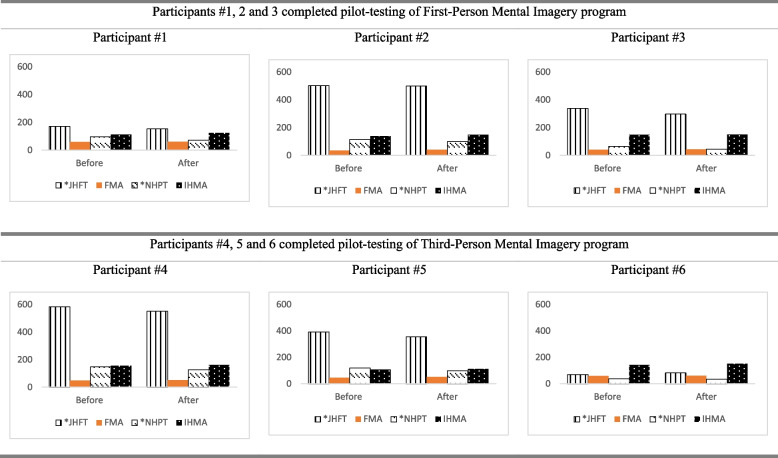
Results of performance-based upper extremity and hand function

**Fig. 2:**
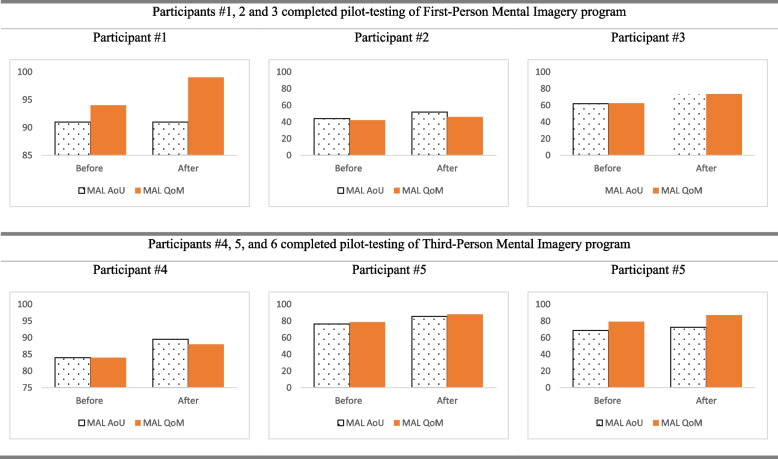
Results of self-perceived upper extremity and hand function

**Fig. 3:**
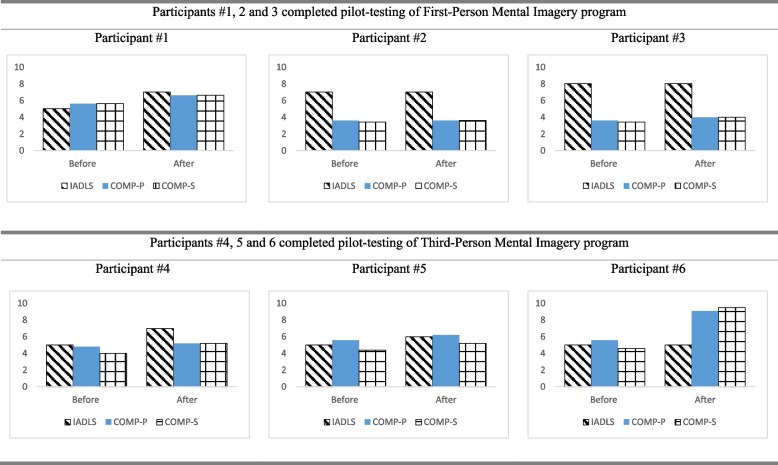
Results of self-perceived daily functions

**Fig. 4:**
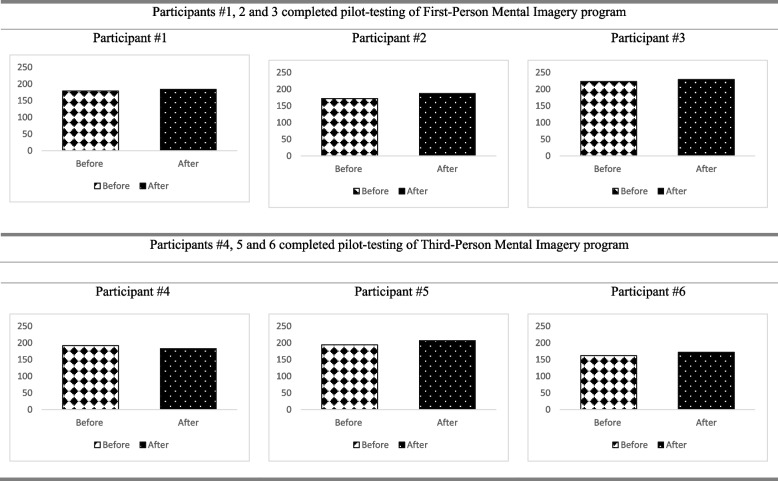
Results of stroke specific quality of life questionnaire

Participant #1 was a 62-year-old male with right-sided weakness following an intracranial hemorrhage 6 months ago. He is right-hand dominant. He attended regular occupational therapy and physiotherapy twice a week. After attending four treatment sessions in the pilot study, all outcome measures of the upper extremity and hand function showed a positive trend towards improvement. The time for completing JTHFT and NHPT was slightly reduced (Fig. 1). There was a slight improvement in FMA, IHMA, MAL-AOU, and MAL-QOM (Figs. 1 and 2). After attending four sessions of FPMI intervention, there was an improvement of IADLS, COPM-P, and COPM-S (Fig. 3). A positive trend of improvement was observed in SSQOL (Fig. 4).

#### Participant #2 (completed the FPMI program)

Participant #2 was a 52-year-old female with right-sided weakness resulting from left cortical infarction 28 months ago. She is right-hand dominant. She attended occupational therapy twice a week. After attending four treatment sessions of FPMI, all the upper extremity and hand function measures showed a positive trend towards improvement. The time for completing JTHFT and NHPT were slightly reduced (Fig. 1). There was a slight improvement in FMA, IHMA, MAL-AOU, and MAL-QOM (Figs. 1 and 2). IADLS and COPM-P were the same after the four sessions of FPMI program (Fig. 3). However, a slight improvement in COPM-S indicated a positive trend towards improvement in daily functions (Fig. 3). There was also a positive trend towards improvement from baseline to post-program assessment in SSQOL (Fig. 4).

#### Participant #3 (completed the FPMI program)

Participant #3 was a 67-year-old female with right-sided weakness resulting from a left ischemic stroke 11 months ago. She is right-hand dominant. She attended occupational therapy twice a week. At the end of four treatment sessions in 2 weeks, all the upper extremity and hand function measures showed a positive trend towards improvement. The time for completing JTHFT and NHPT was slightly reduced (Fig. 1). There was a slight improvement in FMA, IHMA MAL-AOU, and MAL-QOM (Figs. 1 and 2). There was also a slight improvement in IADLS, COPM-P, and COPM-S, which indicated a slight improvement in self-perceived daily functions (Fig. 3). A positive trend towards improvement was noted in SSQOL (Fig. 4).

The individual results of all three participants in the FPMI program showed a positive trend towards improvement in the performance-based and self-perceived upper extremity and hand function. Two participants in the FPMI group demonstrated a slight improvement in self-perceived activities of daily functions as measured by IADLS and perceived performance and satisfaction of daily tasks measured by COPM-P and COPM-S, respectively. However, Participant #2, a 46-year female with right side weakness, did not show progress in IADLS and COPM-P assessments but slight progress in COPM-S. This result can be interpreted as either these assessments were too simple and therefore had a ceiling effect, or a more extended period of training may be necessary for a positive outcome. Another possibility may be a disparity of the results between the performance-based and perceived outcome measures. A case study conducted to identify the effectiveness of CIMT also reported that irrespective of the remarkable progress of functional tasks, the participants did not show improvement in perceived performance [33]. Gillot, Holder-Walls [34] also reported that decreased progress of perceived outcome measures reflected an increased expectation of functional ability.

While two participants in the FPMI group demonstrated improved quality of life, Participant #2 showed a slight decrease. A possible explanation for this finding is that the perceived measurements were subjective to individual cognitive and emotional status [35]. Participant #2 was the youngest (46 years) in this cohort, and she may have set high expectations for her quality of life with the new intervention and may not have achieved the level expected in two weeks of practice.

#### Particpant #4 (completed the TPMI program)

Participant #4 was a 46-year-old female with left-sided weakness resulting from right frontoparietal infarction 6 months ago. She is right-hand dominant. After attending four treatment sessions in 2 weeks, all the upper extremity and hand function measures showed a positive trend towards improvement. Compared to before and after the intervention, the time for completing JTHFT and NHPT was slightly reduced (Fig. 1). There was a slight improvement in FMA, IHMA, MAL-AOU, and MAL-QOM (Figs. 1 and 2). There was an improvement in IADLS, COPM-P, and COPM-S after 2 weeks of the TPMI program (Fig. 3). However, there was a slight decrease in the SSQOL score after the intervention (Fig. 4).

#### Participant #5 (completed the TPMI program)

Participant #5 was a 53-year-old female with right-sided weakness resulting from right frontoparietal infarction 12 months ago. She is right-hand dominant. She attended occupational therapy and physiotherapy twice a week. After attending four treatment sessions in 2 weeks, all the upper extremity and hand function measures showed a positive trend of improvement. The time for completing JTHFT and NHPT was slightly reduced (Fig. 1). There was a slight improvement in FMA, IHMA, MAL-AOU, and MAL-QOM (Figs. 1 and 2). IADLS, COPM-P, and COPM-S demonstrated a slight improvement (Fig. 3). There was a positive trend towards improvement in SSQOL (Fig. 4).

#### Participant #6 (completed the TPMI program)

Participant #6 was a 60-year-old male with right-sided weakness resulting from left cortical infarction nine months ago. He is right-hand dominant. He attended occupational therapy and physiotherapy twice a week. After attending four treatment sessions, all performance-based and self-perceived upper extremity and hand function measures showed a positive trend of improvement. The time for completing JTHFT and NHPT was slightly reduced (Fig. 1). There was a slight improvement in FMA, IHMA, MAL-AOU, and MAL-QOM (Figs. 1 and 2). IADLS was the same after four sessions of the TPMI program (Fig. 3). However, there was a slight improvement in COPM-S and COPM-P (Fig. 3). A positive trend of improvement in SSQOL was observed (Fig. 4).

### General discussion

Mental imagery has demonstrated a beneficial role in improving daily function [19, 36, 37] and hand function [20, 38] in people with stroke. The benefits of first-person imagery from a self-generated image on the consciousness of motor function [11, 39] and third-person imagery from an observed action in enhancing the subjective sense of body ownership [39–41] from the neuroscience perspective have also been demonstrated. Our study demonstrated the potential benefits of the FPMI and TPMI programs in promoting hand function for people with stroke living in the community. Currently, no study has reported the use of first-person and third-person imagery in rehabilitation. Only one study looked at the use of internal and external perspectives rather than first-person and third-person imagery in stroke [10]. There are few studies reporting the use of first-person and third-person imagery in other practice, for example, on sports skills [12]. As there appears to be unique benefits in these two perspectives of imagery, it is essential to research their use in people with stroke. 

This study described the development of the FPMI and TPMI programs specifically targeting hand function for people with stroke living in the community and pilot-testing the intervention protocols. The study found that both programs appeared viable for people with stroke living in the community. The selection criteria adopted in the pilot-testing were appropriate. Participants were able to follow the instructions and completed the intervention sessions within the specified time. Participants were able to complete all outcome measures used without significant issues. Suggestions had been provided regarding offering short breaks for taking water during the thinking aloud component and keeping the video footage about 2 ft. away during the TPMI program. Our results were in alignment with previous studies [19, 20, 36–38] and showed a positive trend towards improvement in hand and daily function after 2 weeks (four sessions) of the programs. The therapist delivering the intervention was able to adhere to the intervention protocols. Therapists were able to conduct the intervention protocol of both the FPMI and TPMI programs as planned. As suggested by Nilsen, Gillen [10] and Page, Dunning [20], the results of this study also showed that a 45-min duration for the intervention session was sufficient for adults with stroke living in the community. Our study was limited by the recruitment methods and the small number of participants in pilot testing. Full programs were not administered. Therefore, conducting a full randomized controlled trial is recommended to verify the effectiveness of the FPMI and TMPI programs. Futher research may consider exploring the benefits of FPMI and TPMI on people with different areas of brain lesions.

## Conclusion

This study examined the development of the FPMI and TPMI intervention programs and their viability by trialling the two programs with six adults with stroke living in the community. It provided preliminary evidence that these MI programs and outcome measures can be feasibly implemented for adults with stroke living in the community. Both the FPMI and TPMI programs positively impacted performance-based upper extremity and hand function, self-perceived upper extremity and hand function, self-perceived daily functions and quality of life. Based on the findings, the study protocol for the FPMI and TPMI intervention programs for the future randomised controlled trial was developed. Experience gathered in this pilot-test enables development of a realistic plan for future trials in relation to participant recruitment, training of therapists on the intervention delivery, and outcome measures.

## Supplementary Information

**Additional file 1: Appendix I.** Intervention programs.

**Additional file 2: Appendix II.** Outcome measures used in the pilot-testing.

## Data Availability

The datasets used and/or analysed during the current study are available from the corresponding author on reasonable request.

## Abbreviations

COPM-P: Canadian Occupational Performance Measure—Performance
COPM-S: Canadian Occupational Performance Measure—Satisfaction
FPMI: First-Person Mental Imagery
FMA: Fugl Meyer Assessment
JTHFT: Jebsen-Taylor Hand Function Test
IADLS: Lawton Instrumental Activities of Daily Living Scale
IHMA: In-Hand Manipulation Assessment
MAL-AOU: Motor Activity Log – Amount of Use
MAL-QOM: Motor Activity Log – Qualtiy of Movement
NHPT: Nine Hole Peg Test
SSQOL: Stroke Specific Quality of Life Scale
TPMI: Third-Person Mental Imagery
