# Supplementary material for: Development and feasibility of first- and third-person motor imagery for people with stroke living in the community
Source: Pilot Feasibility Stud. 2023 Mar 3;9:33. doi: 10.1186/s40814-023-01263-9 (PMC9983213; doi:10.1186/s40814-023-01263-9)
Supplement: Supplementary file 1 — Additional file 1: Appendix I. Intervention programs. [file 40814_2023_1263_MOESM1_ESM.docx]

**Additional file 1: Appendix I - Intervention Programs**

The First-Person Mental Imagery (FPMI) and Third-Person Mental Imagery (TPMI) programs were developed according to the recommendations from the previous literature and adapted to incorporate 12 daily hand tasks. Both the FPMI and TPMI programs were derived from the previously developed programs for adults with stroke (Liu et al., 2004; Nilsen et al., 2012).

Structure of the Programs

The full intervention programs are conducted 45 minutes a day, two sessions a week for six weeks. Participants practise three daily hand tasks each day (Table A). These tasks are categorised and arranged in ascending order based on the complexity of in-hand manipulation domains involved (Table B).

Table A. Structure of the Programs

| Stages of the program | Session number | Daily hand tasks to be practiced |
| --- | --- | --- |
| 1  (Weeks 1 and 2) | 1 | 1, 2, 3 |
|  | 2 | 1, 2, 4 |
|  | 3 | 1, 3, 4 |
|  | 4 | 2, 3, 4 |
| 2  (Weeks 3 and 4) | 5 | 5, 6, 7 |
|  | 6 | 5, 6, 8 |
|  | 7 | 5, 7, 8 |
|  | 8 | 6, 7, 8 |
| 3  (Weeks 5 and 6) | 9 | 9, 10, 11 |
|  | 10 | 9, 10, 12 |
|  | 11 | 9, 11, 12 |
|  | 12 | 10, 11, 12 |

Table B. Hand Tasks Categorised Based on Complexity Levels (Ascending Order)

| Complexity level | Daily hand tasks |
| --- | --- |
| Level 1 | 1. Buttoning a shirt |
|  | 1. Putting toothpaste on a toothbrush |
|  | 1. Using a mobile phone |
|  | 1. Wearing a wristwatch |
| Level 2 | 1. Using a comb |
|  | 1. Taking money from a purse |
|  | 1. Working with a TV remote |
|  | 1. Putting a letter into an envelope |
| Level 3 | 1. Writing with a pen |
|  | 1. Eating a biscuit |
|  | 1. Opening a padlock |
|  | 1. Unpacking groceries |

# *Procedures for the FPMI program*

The program incorporates first-person mental imager and actual practice of the 12 daily hand tasks.

Step 1: The therapist provides instruction on the task and the associated task steps

Step 2: The therapist provides the cue cards on the task steps and the participant reads the task steps

Step 3: The therapist asks the participant to perform the task once (step-by-step) [Therapist will guide the participant to perform the exact task steps if required]

Step 4: The therapist asks the participant to read the task steps and imagine performing the task

Step 5: The participant imagines performing the task with eyes closed. The participant describes the steps as he/she is imagining them (think aloud)

Step 6: The therapist gets feedback from the participant on any difficulties in practising imagery

Step 7: The participant engages into the actual practice of the task

# *Procedures for the TPMI program*

The program incorporates third-person mental imagery and actual practice of the 12 daily hand tasks.

Step 1: The therapist provides instruction on the task and the associated task steps

Step 2: The therapist provides the cue cards on the task steps and the participant reads the task steps

Step 3: Participant watches a video of the task performed by someone else.

Therapist is seated alongside with the participant on the paretic side.

Step 4: The therapist asks the participant to read the task steps and imagine the presenter performing the task that was just shown in the video

Step 5: The participant imagines the presenter performing the task with eyes closed. The participant describes the steps as he/she is imagining them (think aloud)

Step 6: The therapist gets feedback from the participant on any difficulties in practising imagery

Step 7: The participant engages into the actual practice of the task
