# Supplementary material for: Development and feasibility of first- and third-person motor imagery for people with stroke living in the community
Source: Pilot Feasibility Stud. 2023 Mar 3;9:33. doi: 10.1186/s40814-023-01263-9 (PMC9983213; doi:10.1186/s40814-023-01263-9)
Supplement: Supplementary file 2 — Additional file 2: Appendix II. Outcome measures used in the pilot-testing. [file 40814_2023_1263_MOESM2_ESM.docx]

**Additional file 2: Appendix II – Outcome measures used in the pilot-testing**

Jebsen-Taylor Hand Function Test (JTHFT) (Jebsen et al., 1969)

| Use: | Evaluates hand function. |
| --- | --- |
| Instrument: | Consists of seven items - 1) writing; 2) turning 5 cards; 3) picking up small objects and placing them in a tin; 4) stacking for draughts pieces on a board; 5) simulated feeding; 6) lifting empty tin; and 7) lifting heavy tin. |
| Measurement: | Objectively measured using a stopwatch. |
| Administration time: | 6 to 30 minutes. |
| Scoring: | Time taken to complete the task, recorded in seconds. Less time indicates better hand function. |
| Test-retest reliability: | Good, r = .69 to .73 (Beebe & Lang, 2009). |
| Validity | Satisgactory construct validity – Moderate significant correlations with the Wolf Motor Function Test, r = .34 to .68 (Berardi et al., 2019). |

Fugl-Meyer Assessment of Motor Recovery after Stroke (FMA) (Fugl-Meyer et al., 1975)

| Use: | Evaluates motor functioning, balance, sensation and joint functioning in patients with post-stroke hemiplegia. |
| --- | --- |
| Instrument: | Paper based test with five domains assessed include 1) motor function (upper extremity maximum score = 66; lower extremity maximum score = 34); 2) sensory function (maximum score = 24); 3) balance (maximum score = 14); 4) joint range of motion (maximum score = 44); and 5) joint pain (maximum score = 44). |
|  | Subscales can be administered without using the full test (in this study motor function of the upper limb was assessed). |
| Administration time: | 30 minutes (shortened versions > 10 minutes). |
| Scoring: | Scale of 0 to 2. Lowest score 0 indicating poor performance, highest score 226 indicating the best performance. |
| Test-retest reliability: | Excellent, ICC > .95 (Platz et al., 2005) |
| Validity | Correlated moderately highly with the Hemispheric Stroke Scale, a measure of impairment, rho = .66 – .69 (Platz et al., 2005) |

Nine Hole Peg Test (NHPT) (Mathiowetz et al., 1985)

| Use: | Evaluates finger dexterity. |
| --- | --- |
| Instrument: | Consists of pegs, nine-hole pegboard and a stopwatch. |
| Measurement: | Objectively measured using a stopwatch. |
| Administration time: | < 1 minute. |
| Scoring: | Based on the time taken to complete the test activity, recorded in seconds. Less time indicating better finger dexterity. |
| Test-retest reliability: | Excellent, ICC = .83 to .99 (Ekstrand et al., 2016) |
| Validity | Satisfactory convergent validity, rho = .68 with Sollerman Hand Function Test (Ekstrand et al., 2016) |

In-Hand Manipulation Assessment (IHMA) (Klymenko et al., 2018)

| Use: | Evaluate in-hand manipulation skills. |
| --- | --- |
| Instrument: | Consists of 20 hand tasks. |
| Areas of assessment: | 1) finger-to-palm and palm-to-finger translation; 2) shift; 3) simple rotation; 4) complex rotation; and 5) stabilisation. |
| Administration time: | 15 minutes. |
| Scoring: | Skill performance average 0 - 4 (highest score indicating no difficulty). |
| Inter-rater reliability: | Not reported. |
| Validity | Content validity established (Klymenko et al., 2018). |

Motor Activity Log (MAL) (Duncan et al., 2003)

| Use: | Evaluates Quality of Movement (QOM) and Amount of Use (AOU). In this study MAL 30 with 30 functional tasks was used. |
| --- | --- |
| Instrument: | Paper based test with 30 daily functional tasks. |
| Areas of assessment: | Amount of Use (AOU) and Quality of Movement (QOM) scales on the 30 daily functional tasks. |
| Administration time: | Approximately 20 minutes. |
| Scoring: | Semi structured interview scale ranges from 0 (never used) to 5 (same as pre-stroke).  0 = low amount of use and low quality of movement  300 = Highest AOU and QOM. |
| Test-retest reliability: | Excellent for both AOU and QOM, ICC = .99 (Pereira et al., 2012). |
| Validity | Fair to moderate construct validity with the 16-hole peg test (r = −.64 to −.67) (Hammer & Lindmark, 2010) |

Lawton Instrumental Activities of Daily Living Scale (IADL) (Lawton & Brody, 1969)

| Use: | Evaluates instrumental activities of daily living. |
| --- | --- |
| Instrument: | Paper based test consists of eight tasks. |
| Areas of assessment: | 1) telephone use; 2) shopping; 3) food preparation; 4) housekeeping; 5) laundry; 6) transportation; 7) medical management; and 8) financial management. |
| Administration time: | 10 – 15 minutes. |
| Scoring: | Scale of 0 – 1 in each item. Total score of 0 indicating dependent in the eight tasks, 8 indicating independent. |
| Test-retest reliability: | Excellent, ICC = .99 (Tong & Man, 2002). |
| Validity | Good construct validity – demonstrated a one-factor structure (Tong & Man, 2002). |
|  |  |

Canadian Occupational Performance Measure (COPM) (Law et al., 1998)

| Use: | Evaluates a person’s self-perception of occupational performance and satisfaction with performance. |
| --- | --- |
| Instrument: | Paper based assessment involves a 5-step process nested within a semi-structured interview. |
| Areas of assessment: | 1) self-perceived performance; 2) satisfaction on performance. |
| Administration time: | 6 - 30 Minutes |
| Scoring: | 10-point ordinal scale, 1 indicating poor performance and satisfaction, 10 indicating the best performance and satisfaction. |
| Test-retest reliability: | Excellent, r = 0.89 for performance and 0.88 for satisfaction (Cup et al., 2003). |
| Validity: | Poor to fair criterion-related validity between the COPM and Functional Independence Measure (r = −0.14 to 0.38) (Chan & Lee, 2006). |

Stroke Specific Quality of Life scale (SS-QOL) (Williams et al., 1999)

| Use: | Evaluates health-related quality of life. |
| --- | --- |
| Instrument: | Paper based test with 12 domains. |
| Areas of assessment: | 1) energy; 2) family roles; 3) language; 4) mobility; 5) mood; 6) personality; 7) self-care; 8) social roles; 9) thinking; 10) upper extremity function; 11) vision; and 12) work/productivity. |
| Administration time: | 10-15 minutes. |
| Scoring: | Scale of 1-5 with overall.  Lowest score 49 indicating total help needed, highest score 245 indicating no help needed. |
| Test-retest reliability: | Excellent, rho = .65 - .99 (Muus et al., 2007). |
| Validity: | Discriminant validity established (Muus et al., 2007). |

Beebe, J. A., & Lang, C. E. (2009, Jun). Relationships and responsiveness of six upper extremity function tests during the first six months of recovery after stroke. *Journal of Neurologic Physical Therapy, 33*(2), 96-103. <https://doi.org/10.1097/NPT.0b013e3181a33638>

Berardi, A., Saffioti, M., Tofani, M., Nobilia, M., Culicchia, G., Valente, D., Servadio, A., & Galeoto, G. (2019). Internal consistency and validity of the Jebsen-Taylor hand function test in an Italian population with hemiparesis. *NeuroRehabilitation, 45*(3), 331-339. <https://doi.org/10.3233/nre-192867>

Chan, C. C. H., & Lee, T. M. C. (2006). Validity of the Canadian occupational performance measure. *Occup Ther Int, 4*(3), 231-249. <https://doi.org/https://doi.org/10.1002/oti.58>

Cup, E. H. C., Scholte op Reimer, W. J. M., Thijssen, M. C. E., & van Kuyk-Minis, M. A. H. (2003, 2003/06/01). Reliability and validity of the Canadian Occupat189ional Performance Measure in stroke patients. *Clinical Rehabilitation, 17*(4), 402-409. <https://doi.org/10.1191/0269215503cr635oa>

Duncan, P. W., Bode, R. K., Min Lai, S., & Perera, S. (2003). Rasch analysis of a new stroke-specific outcome scale: the Stroke Impact Scale. *Archives Of Physical Medicine And Rehabilitation, 84*(7), 950-963.

Ekstrand, E., Lexell, J., & Brogårdh, C. (2016, 2016/10/01/). Test−Retest Reliability and Convergent Validity of Three Manual Dexterity Measures in Persons With Chronic Stroke. *PM&R, 8*(10), 935-943. <https://doi.org/https://doi.org/10.1016/j.pmrj.2016.02.014>

Fugl-Meyer, A. R., Jääskö, L., Leyman, I., Olsson, S., & Steglind, S. (1975). The post-stroke hemiplegic patient. 1. a method for evaluation of physical performance. *Scandinavian journal of rehabilitation medicine, 7*(1), 13-31.

Hammer, A. M., & Lindmark, B. (2010, 2010/01/01). Responsiveness and validity of the Motor Activity Log in patients during the subacute phase after stroke. *Disability And Rehabilitation, 32*(14), 1184-1193. <https://doi.org/10.3109/09638280903437253>

Jebsen, R. H., Taylor, N., Trieschmann, R., Trotter, M. J., & Howard, L. A. (1969). An objective and standardized test of hand function. *Archives Of Physical Medicine And Rehabilitation, 50*(6), 311.

Klymenko, G., Liu, K. P., Bissett, M., Fong, K. N., Welage, N., & Wong, R. S. (2018). Development and initial validity of the in‐hand manipulation assessment. *Australian Occupational Therapy Journal*.

Law, M. C., Baptiste, S., Carswell, A., McColl, M. A., Polatajko, H., & Pollock, N. (1998). *Canadian occupational performance measure*. Canadian Association of Occupational Therapists Ottawa ON.

Lawton, M. P., & Brody, E. M. (1969). Assessment of older people: self-maintaining and instrumental activities of daily living. *The gerontologist, 9*(3_Part_1), 179-186.

Mathiowetz, V., Weber, K., Kashman, N., & Volland, G. (1985). Adult norms for the nine hole peg test of finger dexterity. *OTJR: Occupation, Participation and Health, 5*(1), 24-38.

Muus, I., Williams, L. S., & Ringsberg, K. C. (2007, 2007/07/01). Validation of the Stroke Specific Quality of Life Scale (SS-QOL): test of reliability and validity of the Danish version (SS-QOL-DK). *Clinical Rehabilitation, 21*(7), 620-627. <https://doi.org/10.1177/0269215507075504>

Pereira, N. D., Ovando, A. C., Michaelsen, S. M., Anjos, S. M., Lima, R. C., Nascimento, L. R., & Teixeira-Salmela, L. F. (2012, Mar). Motor Activity Log-Brazil: reliability and relationships with motor impairments in individuals with chronic stroke. *Arq Neuropsiquiatr, 70*(3), 196-201. <https://doi.org/10.1590/s0004-282x2012000300008>

Platz, T., Pinkowski, C., van Wijck, F., Kim, I.-H., di Bella, P., & Johnson, G. (2005). Reliability and validity of arm function assessment with standardized guidelines for the Fugl-Meyer Test, Action Research Arm Test and Box and Block Test: a multicentre study. *Clinical Rehabilitation, 19*(4), 404-411. <https://doi.org/10.1191/0269215505cr832oa>

Tong, A. Y. C., & Man, D. W. K. (2002, 2002/10/01). The Validation of the Hong Kong Chinese Version of the Lawton Instrumental Activities of Daily Living Scale for Institutionalized Elderly Persons. *OTJR: Occupation, Participation and Health, 22*(4), 132-142. <https://doi.org/10.1177/153944920202200402>

Williams, L. S., Weinberger, M., Harris, L. E., Clark, D. O., & Biller, J. (1999). Development of a stroke-specific quality of life scale. *Stroke, 30*(7), 1362-1369.
